# Isolation of Naturally Induced T-regulatory Cells in Gingival Tissues of Healthy Human Subjects and Subjects with Gingivitis and Chronic Periodontitis

**DOI:** 10.7759/cureus.4283

**Published:** 2019-03-20

**Authors:** Devi Arul, Suresh Rao

**Affiliations:** 1 Periodontics, Sri Ramachandra Medical College and Research Institute, Chennai, IND

**Keywords:** regulatory t cells, ntreg, self tolerance, flow cytometry, chronic periodontitis

## Abstract

Background: The immune mechanism depends on CD4+ T cells for its regular function, and altered T cell function leads to microbial disease progression.

Aim: The present study aimed to determine the role of naturally induced T-regulatory (nTreg) cells (CD4+ CD25+ Fox P3+) in periodontal disease pathogenesis.

Materials and methods: A total of 30 patients attending the out-patient clinic of the Department of Periodontology and Implantology, Faculty of Dental Sciences, Sri Ramachandra University (SRU), Chennai, India were recruited for the study. They were categorized in three groups as healthy individuals, individuals with chronic gingivitis, and individuals with chronic periodontitis gingival tissues. nTreg (CD4+ CD25+ Fox P3+) cells were isolated using flow cytometry. Different conjugated, isolated cells were then gated in the order of CD4+, CD25+, and Fox P3+ cells.

Results: The results of our study showed an increase in the proportions of Treg cells in individuals with chronic periodontitis compared to individuals with gingivitis and healthy individuals.

Conclusion: Further elucidation of cellular and molecular processes underlying Treg cells will help unravel the complexity behind periodontal disease pathogenesis besides paving the way in developing newer treatment strategies.

## Introduction

Periodontal disease is a multifactorial disease where infection and its sequel, inflammation and immunity, play a critical role in disease pathogenesis [1]. Though the indispensable role of microorganisms leads to the initiation of disease, the progression and severity of the disease are due to the exaggerated host immune response. Periodontal disease is characterized by periods of exacerbation interspersed with periods of remission and is also regarded as a healing lesion. Available evidence suggests T-regulatory (Treg) cells as key anti-inflammatory cells that are critically involved in limiting the inflammatory response [2]. These cells are described as those T cells that have escaped deletion in the thymus and later become anergic or get suppressed by various mechanisms [3]. Treg cells can be defined as a group of T-cell population that functionally suppresses an immune response by influencing the activity of another cell type [4].

A major challenge in immunology and medicine was to determine the unresponsiveness of the adaptive immune system to self-antigens (that is, immunological self tolerance), which is now well established [5]. The immune mechanism has to deal with an array of inflammatory signals during long established infections like chronic periodontitis. There may be self control mechanisms where regulation of effector cells may take place. To assess this control mechanism, our present study was carried out to determine the presence of naturally induced Treg (nTreg) cells in states of gingival health, gingivitis, and chronic periodontitis.

## Materials and methods

Study population

**Figure 1:**
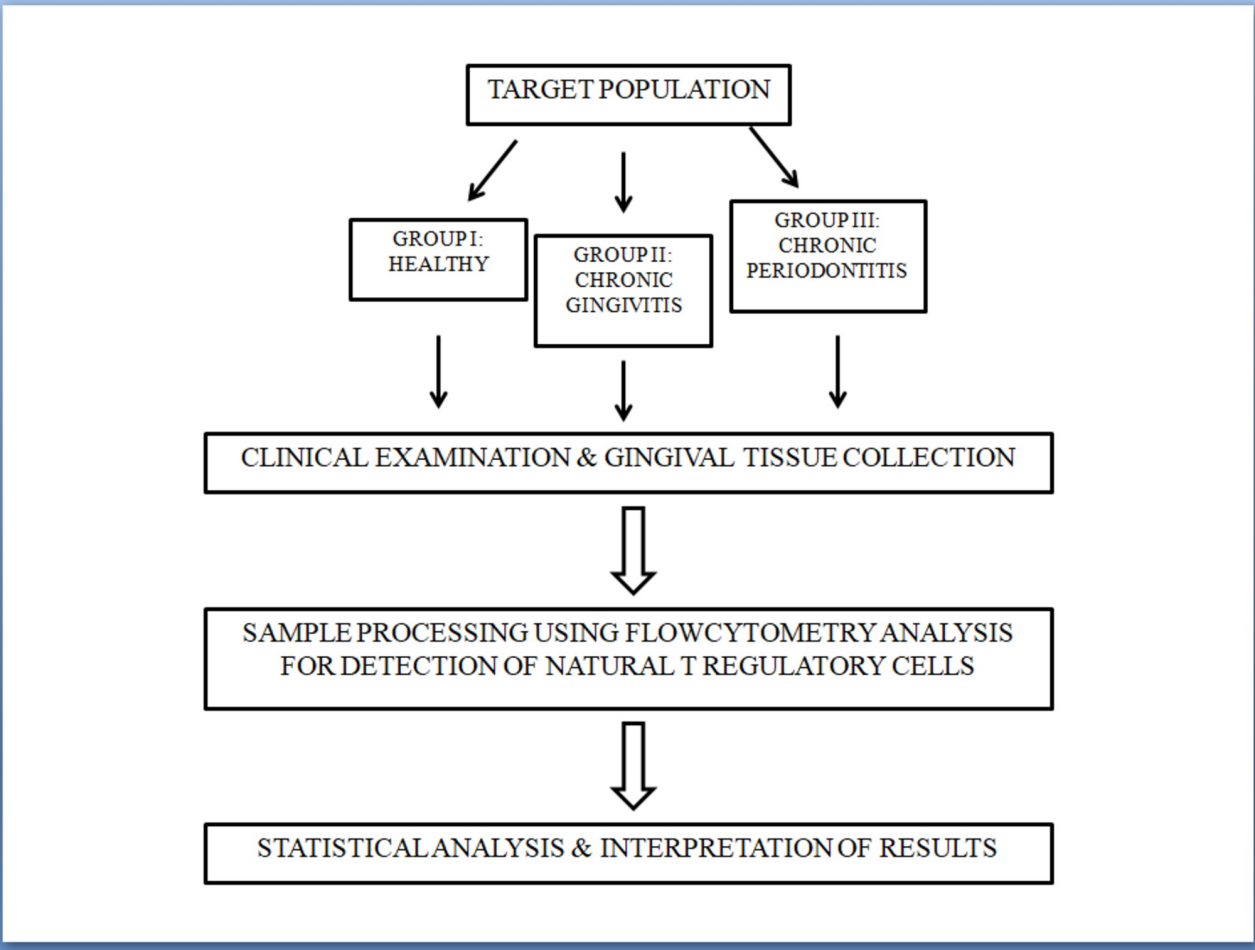
Selection of cases for the study.

This study was approved by the institutional ethics committee of Sri Ramachandra University, Chennai, India. A written informed consent was obtained from all the patients included in the study. The study comprised of three groups namely healthy individuals (group I; n=10), chronic generalized gingivitis (group II; n=10), and chronic periodontitis (group III; n=10) (Figure 1).

Patients with clinically healthy gingiva with absence of bleeding on probing were included in group I. Patients with chronic generalized gingivitis with clinical signs of gingival inflammation with no evidence of clinical attachment loss or radiographic bone loss were included in group II. Patients with more than 20 teeth with clinical attachment loss >1-2 mm and radiographic evidence of bone loss were included in group III [6].

The exclusion criteria for all the groups were presence of any systemic disease, use of tobacco in any form, previous history of periodontal therapy, and those who had taken antibiotics or analgesics recently. Pregnant and lactating women were excluded from the study.

Healthy gingival tissues were obtained during crown lengthening procedures or therapeutic orthodontic extractions; chronic generalized gingivitis samples were obtained during gingivectomy procedures, and chronic generalized periodontitis tissue samples were obtained from hopeless teeth prior to extraction procedure.

Reagents used and flow cytometry analysis

The following markers that are specific for regulatory T cells were used in the analysis.

Fluorochrome conjugated monoclonal antibodies (Bd Biosciences, San Diego, CA, USA):

· 555348 PE-Cy 5 Mouse Anti-Human CD4

· 555432 PE Mouse Anti-Human CD25

· 561181 Alexa Flour 488 Mouse Anti-Human Fox P3

· Fox P3 buffer kit for fixing and permeabilization

o 51-9005451 (Fox P3 buffer A)

o 51-9005450 (Fox P3buffer B)

· 554656 BD Stain buffer (Fetal bovine Serum)

· Phosphate Buffered Saline pH 7.4

All samples were thawed on ice and mononuclear cell suspensions were prepared using an ultrasonic processor with 100-200 µl of phosphate buffered saline (PBS). The prepared cells were centrifuged at 1500 rpm for three minutes following which the supernatant was discarded. Then 200 µl of PBS was added to the pellet and again centrifuged at 1500 rpm for three minutes.

Thus a final cell pellet was obtained to which 100 µl of PBS, 20 µL of CD4 PECY5, 20 µl of CD25PE, and 100 µl of stain buffer were added. The samples were then incubated on ice for an hour in the dark following which they were again centrifuged. For intracellular staining, a final workable solution of Fox P3 buffer C was obtained by diluting Fox P3 buffer A and B according to the instructions of the manufacturer.

**Figure 2:**
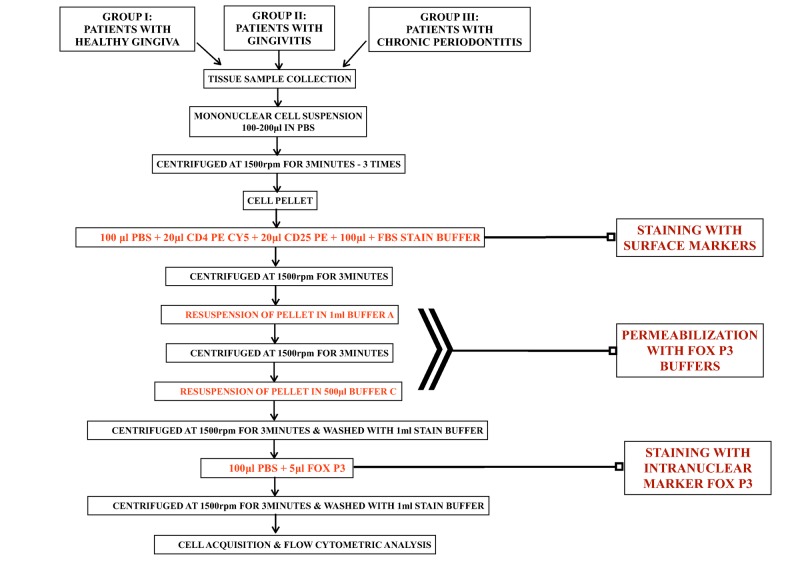
Flow diagram for tissue processing.

Following this, 1 ml of Buffer ‘A’ was added to the pellet and incubated for 10 minutes at room temperature. The samples were then washed with a stain buffer and underwent centrifugation. The obtained cell pellets were re-suspended in 500 µl of buffer ‘C’ and incubated for 30 minutes at room temperature. After a wash with 1 ml of stain buffer, 100 µ PBS and 5 µl of Fox P3 Alexa Flour 488 were added to the cell pellets and incubated for 30 minutes at room temperature. The prepared samples were re-suspended in sheath fluid, following which they were transferred to a fluorescence-activated cell sorting (FACS) tube and used for cell acquisition and flow cytometry analysis (Figure 2).

The gating of the cells in the gingival tissue was done for the lymphocyte region and the fluorescence of these samples was compared with the unstained sample. A total of 25,000 cells were acquired from each sample and the proportions of CD4, CD25, and Fox P3 were gated using the software.

**Figure 3:**
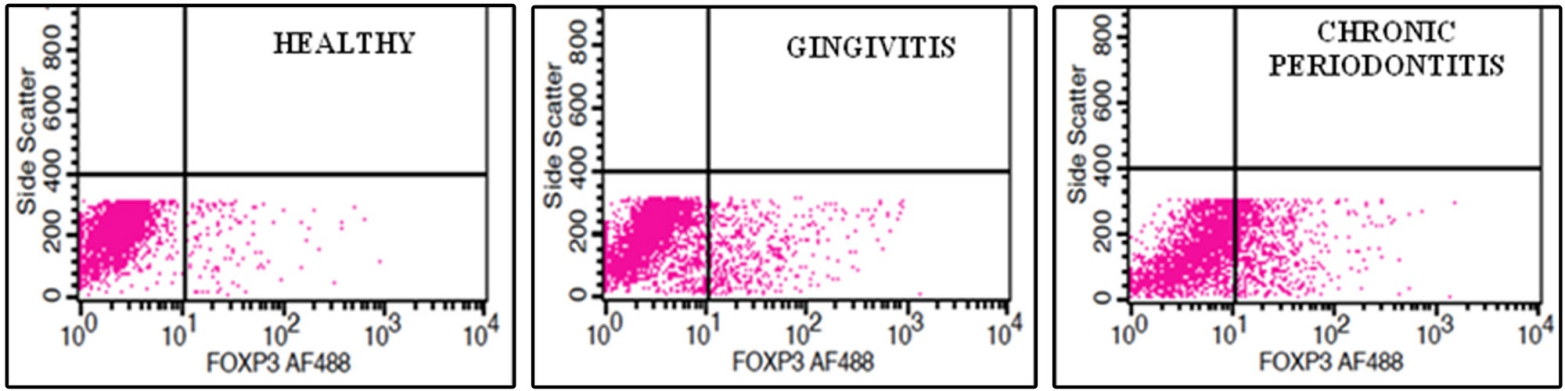
Plot diagram showing the gating of CD4+ CD25+ Fox P3+ cells in healthy, gingivitis and chronic periodontitis samples, respectively.

From the gated regions, the percentage of natural induced T regulatory cells (CD4+ CD25+ FOX P3+) in the three groups (healthy, gingivitis, and chronic periodontitis) were assessed (Figure 3) and their significant differences were estimated using commercially available Statistical Package for the Social Sciences (SPSS, IBM Corp., USA) software.

## Results

A total of 30 individuals were included in this study. They were divided into three groups based on specific inclusion and exclusion criteria as healthy individuals, patients with gingivitis, and patients with chronic periodontitis. The demographic details are summarized in Table 1. There were four females and six males in the healthy group, three females and seven males in the gingivitis group, and two females and eight males in the chronic periodontitis group. The age range of the subjects in the control group was 21-30 years (mean age 24.5 ± 7.74 years), the age range of the gingivitis group was 23-35 years (mean age 27.6 ± 8.72 years), and that of the chronic periodontitis group was 36-58 years (mean age 49.4 ± 15.63 years). The mean Simplified Oral Hygiene Index (OHI (S)) score, probing depth, and clinical attachment level (CAL) are depicted as mean ± standard deviation in Table 1.

**Table 1:** Demographic data of the study groups.

GROUP		I HEALTHY	II GINGIVITIS	III CHRONIC PERIODONTITIS
Number of Subjects		10	10	10
Age (in years depicted as mean ± standard deviation)		24.5±7.74	27.6±8.72	49.4±15.63
Gender	Female	4	3	2
	Male	6	7	8
Mean OHI.S Score (depicted as mean ± standard deviation)		0.91±0.26	1.77±0.55	4.69±6.95
Clinical Attachment Level (depicted as mean ± standard deviation)		0	0	1.98±0.62

The results of our study showed an increase in the proportions of natural Tregs (CD4+ CD25+ Fox P3+) in the chronic periodontitis group compared to the gingivitis and healthy tissue groups. These values were statistically significant. However the number of natural Tregs (CD4+ CD25+ Fox P3+) was not significantly elevated in the gingivitis group compared to the healthy tissue group.

All the samples were stained initially for surface markers such as CD4 and CD25 followed by intracellular staining with Fox P3. The cells that co-expressed all the three markers (CD4, CD25, and Fox P3) were quantified as natural Tregs. ANOVA, post hoc test, and T-test were performed to analyze the results.

Total CD4+ CD25+ Fox P3+ cells

**Figure 4:**
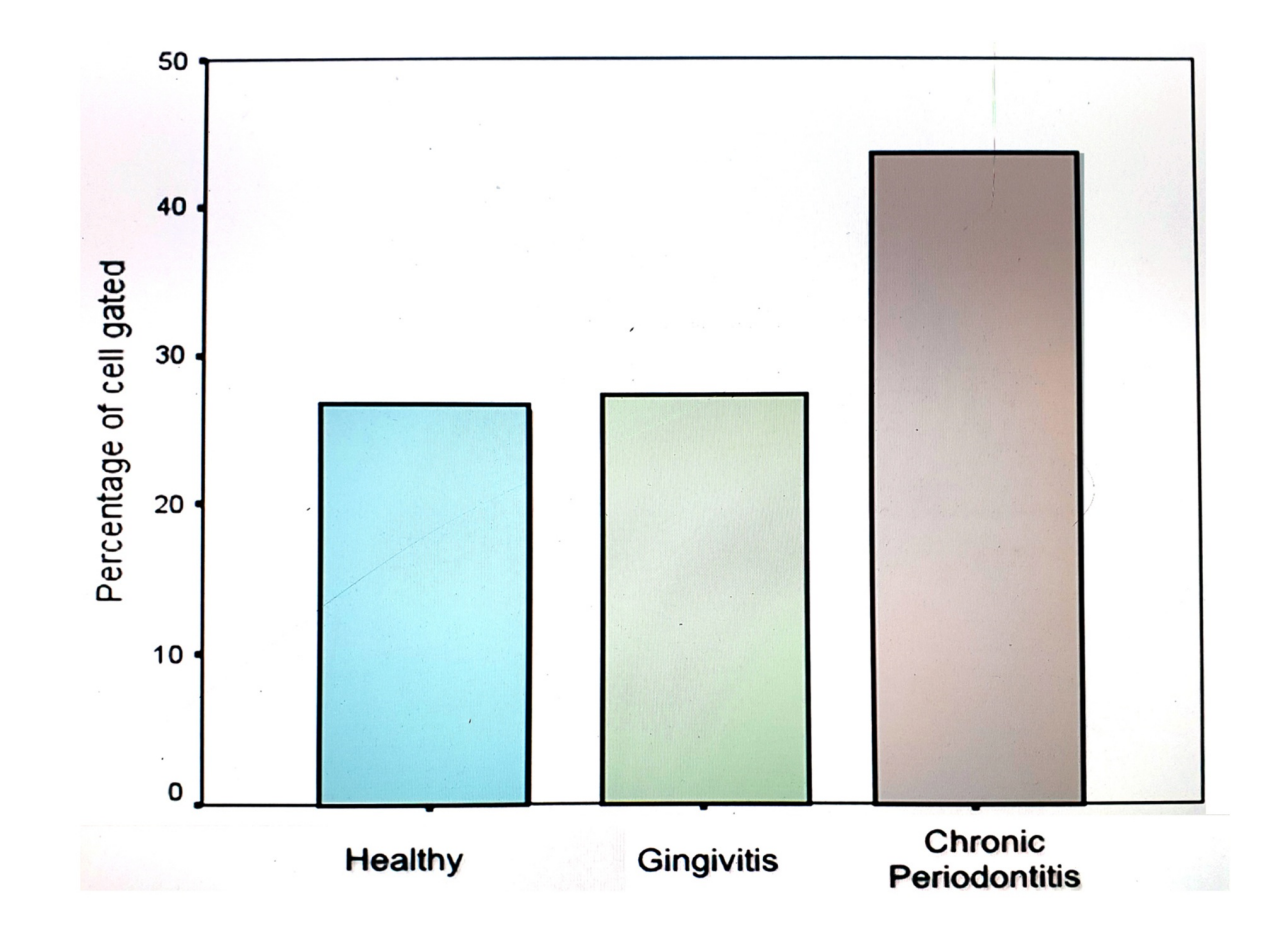
Bar graph showing the comparison of CD4+ CD25+ FOX P3+ T cells in gingival tissue samples of healthy individuals, gingivitis and chronic periodontitis patients.

The results of our study showed an increase in the proportions of natural Tregs (CD4+ CD25+ Fox P3+) in the chronic periodontitis group compared to the gingivitis and healthy tissue groups (Figure 4). These values were statistically significant. However the number of natural Tregs (CD4+ CD25+ Fox P3+) was not significantly elevated in the gingivitis group compared to the healthy group (Tables 2-4).

**Table 2:** One way Anova Test - Comparison of gingival tissues in healthy, gingivitis, and chronic periodontitis patients for CD4+ CD25+ FoxP3+ cells NOTE: ** denotes significance: p value less than 0.01.

GROUPS	MEAN± STANDARD DEVIATION	P VALUE
Healthy	25.6171 ± 9.30536	0.001**
Gingivitis	26.3900 ± 6.48849	
Chronic Periodontitis	42.7557 ± 7.59459	

**Table 3:** Post hoc Tests - Tukey HSD: Multiple comparisons - Comparison of mean values of total nTreg (CD4+ CD25+ FoxP3+) cells between the groups in the gingival tissue extracts. NOTE: *The mean difference is significant at the 0.05 level. HSD - honestly significant difference

GROUP	GROUP	SIG.
Healthy	Gingivitis	.982
	Periodontitis	.003**
Gingivitis	Healthy	.982
	Periodontitis	.002**
Periodontitis	Healthy	.003**
	Gingivitis	.002**

**Table 4:** T-test: Group statistics Comparison of mean values of total nTreg (CD4+ CD25+ FoxP3+) cells between the groups in the gingival tissue extracts.

	GROUP	N	MEAN	STD. DEVIATION	STD. ERROR MEAN	P VALUE
Percentage of cells gated	Healthy	10	26.3900	6.48849	2.45242	0.860 (NS)
	Gingivitis	10	25.6171	9.30536	3.51710	
	Healthy	10	26.3900	6.48849	2.45242	0.001**
	Periodontitis	10	42.7557	7.59459	2.87049	
	Gingivitis	10	25.6171	9.30536	3.51710	0.003**

## Discussion

Periodontal disease has inflammation as the basis with many confounding factors and hence is referred to as a multifactorial disease. It is a result of host-microbial interaction where the inflammatory component leads to major destruction. Some clarity has been obtained in the pathogenesis of the disease after decades of research, but it is still far from a clear picture. An immediate acute inflammation, innate immunity, and adaptive immunity are the three different ways a host can respond [7]. A pool of undifferentiated pre-lymphocytes from hematopoietic stem cells after many transitions stay centrally as nTreg cells. These cells after stringent selection reach the peripheral structures. In the periphery they can differentiate into effectors factors or persist as nTregs or can be made more active induced Tregs (iTregs). Of the three, nTreg is the most immunosuppressive followed by iTreg, but the maximum tissue destruction follows effector cells [8].

In the present study we used CD4, CD25, and Fox P3 as markers to isolate nTregs from gingival tissues. Nakajima et al. in 2005 demonstrated an increase of nTregs in chronic periodontitis and gingivitis in comparison to healthy groups. An immunohistochemical analysis of CD4, CD25, and CTLA-4 and gene expression analysis of Fox P3, TGF-beta, and IL-10 in gingival biopsies revealed the presence of nTregs [9].

They suggested that nTreg cells and other regulatory T cell populations exist and play a role in periodontal disease. Similarly, in our present study we showed a significant increase in the nTregs in chronic periodontitis.

Cardoso et al. in their study showed similar increase in nTreg cells in chronic periodontitis and concluded that Treg cells are involved in the modulation of local immune responses in chronic lesions [10].

Ernst et al. carried out studies correlating the levels of double positive cells with 'receptor activator of nuclear factor kappa-Β ligand' (RANKL) and reported that Treg cells are inversely proportional to RANKL levels depicting their role in chronic conditions [11]. Interestingly, RANKL levels are influenced by immune and inflammatory factors. So, probably, if nTregs are less, the local immune response could be more effector cell dominant and more destructive as evidenced by these cells.

## Conclusions

The results of our study showed an increase in the proportion of nTregs (CD4+ CD25+ Fox P3+) in the chronic periodontitis group compared to the gingivitis and healthy tissue groups. These values were statistically significant. However, there was a noticeable nTreg cell expression in gingivitis that was not statistically significant. Thus, our study results conclude that nTregs play an important role in the disease progression of periodontitis.
